# The relation between harsh parenting and bullying involvement and the moderating role of child inhibitory control: A population‐based study

**DOI:** 10.1002/ab.22014

**Published:** 2021-12-16

**Authors:** Sara I. Hogye, Pauline W. Jansen, Nicole Lucassen, Renske Keizer

**Affiliations:** ^1^ Department of Public Administration and Sociology Erasmus University Rotterdam Rotterdam the Netherlands; ^2^ Generation R Study Group Erasmus MC University Medical Center Rotterdam Rotterdam the Netherlands; ^3^ Department of Child & Adolescent Psychiatry/Psychology Erasmus MC University Medical Centre Rotterdam Rotterdam the Netherlands; ^4^ Department of Psychology, Education & Child Studies Erasmus University Rotterdam Rotterdam the Netherlands

**Keywords:** bullying involvement, fathers, harsh parenting, inhibitory control, mothers

## Abstract

Harsh parenting has been linked to children's bullying involvement in three distinct roles: perpetrators, targets (of bullying), and perpetrator‐targets. To understand how the same parenting behavior is associated with three different types of bulling involvement, we examined the moderating roles of children's inhibitory control and sex. In addition, we differentiated between mothers’ and fathers’ harsh parenting. We analyzed multi‐informant questionnaire data from 2131 families participating in the Dutch Generation R birth cohort study. When children were three years old, parents reported on their own harsh parenting practices. When children were four, mothers reported on their children's inhibitory control. At child age six, teachers reported on children's bullying involvement. Our results revealed that fathers’, and not mothers’, harsh parenting increased the odds of being a perpetrator. No moderation effects with children's inhibitory control and sex were found for the likelihood of being a perpetrator. Moderation effects were present for the likelihood of being a target and a perpetrator‐target, albeit only with mothers’ harsh parenting. Specifically, for boys with lower‐level inhibitory control problems, mothers’ harsh parenting increased the odds of being a target. In contrast, for boys with higher‐level inhibitory control problems, mothers’ harsh parenting decreased the odds of being a target. Furthermore, for girls with higher‐level inhibitory control problems, mothers’ harsh parenting increased the odds of being a perpetrator‐target. Overall, our results underscore the importance of differentiating by children's cognitive skills and by parent and child sex to fully understand how harsh parenting and bullying involvement are related.

## 1 INTRODUCTION

Up to 30% of children between the ages of five and six are involved in bullying (Jansen, Verlinden, et al., 2012; Perren & Alsaker, 2006). Bullying involvement is commonly defined as the behavior of three groups of peers with an imbalance of power between them: *perpetrators, targets*, and those taking up both roles, who are referred to as *perpetrator‐targets* (Olweus, 1993). Perpetrators are assertive and use aggressive and/or hostile behavior toward relatively powerless peers instrumentally to attain a goal (e.g., social dominance; Perren & Alsaker, 2006; Volk et al., 2014). Their behavior is manifested in physical, verbal, material, relational, or cyber forms. Targets (of bullying) are characterized as submissive, rejected, and withdrawn, who face difficulties with defending themselves during negative peer experiences (e.g., when they are socially excluded from play; Perren & Alsaker, 2006; Volk et al., 2014). Perpetrator‐targets share characteristics of both targets (e.g., being rejected) and perpetrators (e.g., aggression; Volk et al., 2014). Whereas pure perpetrators use goal‐oriented proactive aggression, perpetrator‐targets however often exhibit aggressive and impulsive behavior in response to their victimization (Volk et al., 2014). They might target relatively powerless peers (e.g., by name calling) to restore their social status after being victimized (Choi & Park, 2018).

As children's involvement in bullying can leave detrimental marks in various developmental aspects across childhood, adolescence, and young adulthood (Arseneault et al., 2010; Kretschmer et al., 2017), it is crucial to understand the determinants of bullying involvement. In early childhood, when family members are key role models and main socializing figures (Maccoby, 1992), the first signs of bullying involvement are already visible (Barker et al., 2008). Represented by the social cognitive theory (Bandura, 1989), behavior exhibited during bullying involvement may be learned from observing one's parents. Specifically, parental discipline provides key socializing moments for children's learning of moral reasoning and disengagement (Campaert et al., 2018). For example, children who receive harsh discipline after misbehaving may learn that aggression is an acceptable way to achieve a certain outcome (i.e., getting your way, de Vries et al., 2018). In early childhood, when children have few other social circles that could buffer against adverse modeling influences, harsh parenting is an environmental factor to which children are vulnerable and susceptible (Labella & Masten, 2018). Harsh parenting is characterized by coercive acts, harsh and punitive discipline, and negative emotional affect, such as “yelling, frequent negative commands, name calling, overt expression of anger, and physical threats and aggression” (Chang et al., 2003, p. 2).

Harsh parenting is linked to all three bullying involvement roles. Aggressive and coercive parenting has been associated with bullying perpetration and victimization (Barker et al., 2008; Hipwell et al., 2014; Papanikolaou et al., 2011). In a meta‐analysis, focusing on targets and perpetrator‐targets, Lereya et al. (2013) reported that both were more likely than those uninvolved in bullying to be exposed to harsh parenting. In addition, the meta‐analysis by Nocentini et al. (2019) underscored the associations between harsh parenting on the one hand and traditional and cyber‐forms of bullying and victimization on the other. Although not all studies have assessed linkages between harsh parenting and all three types of bullying involvement roles in one and the same study, overall, there appears to be consensus in the literature that harsh parenting is associated with a higher likelihood of involvement in all three roles of perpetrator, target, and perpetrator‐target.

The present study is driven by our interest in this pattern. How can it be that the same maladaptive parenting practices are linked with all three distinct bullying involvement roles? In this study, we aim to explain the relations between harsh parenting and three bullying involvement roles. We argue that these linkages are conditional upon children's inhibitory control levels.

### 1.1 Child inhibitory control as a moderator

Inhibitory control is part of the self‐regulatory executive function, and refers to the ability to stop or modulate behavioral responses and to control impulses (Gioia et al., 2003). Intact inhibitory control is necessary for suitable social information processing (van Nieuwenhuijzen et al., 2017). Impairments in inhibitory control could lead to challenges in the cognitive steps that are needed for appropriate behavior (van Nieuwenhuijzen et al., 2017), and result in disruptive, impulsive, and physically inappropriate behavior (Gioia et al., 2003). Of the five cognitive steps needed for appropriate behavior, two are highly relevant in the context of our research question: “perception of stimuli” (e.g., interpreting intentions of an interaction partner) and “response selection” (e.g., considering self‐efficacy and consequences; van Nieuwenhuijzen et al., 2017). At the step of perception of stimuli, children with higher levels of inhibitory control problems are more likely to attribute *hostile intent* to ambiguous social situations (Ellis et al., 2009) and more likely to select aggressive responses (van Nieuwenhuijzen et al., 2017).

From experiencing harsh parenting, children may learn that aggression is an acceptable behavior either to *imitate* or to *endure* (Bandura, 1973, 1989; Labella & Masten, 2018). Depending on inhibitory control levels, experiencing harsh parenting could contribute to inappropriate coping, either as imitating learnt aggression (perpetrator behavior) or enduring such aggression (target of bullying) within peer circles. Children may learn from harsh parenting that using aggressive behavior against a relatively powerless person who “deserves” it is appropriate (Campaert et al., 2018). Children who have higher levels of inhibitory control problems may show less inhibition of impulses in response to parental harshness and instead reply with more aggressive and hostile responses (Ziv et al., 2013). The rationale is that children who have higher levels of inhibitory control problems deal with their parents’ harshness by an outward expression of their frustrations, and are more likely to imitate physical or verbal behavior experienced during harsh parenting situations (Bandura, 1973). Consequently, they might be more likely to choose hostile and/or aggressive ways of communication in peer interactions, and to perceive a weaker peer as someone who can be blamed. We therefore hypothesize that for children who have higher levels of inhibitory control problems harsh parenting will predict perpetrator behavior (*H1*).

In contrast, children with relatively lower levels of inhibitory control problems can show sufficient inhibition of their impulses which prevents them from responding aggressively to parental harshness. However, as a result of repeated harsh parenting, the child may develop cognitive schemas that perceive the parent as controlling and threatening and the self as helpless and defeated (family relational schema; Batanova & Loukas, 2014; Perry et al., 2001). Children who can inhibit their impulses to some extent then take up a passive role, by exhibiting “compulsively compliant” responses when they learn that they are “powerless vis‐à‐vis the parent” (Perry et al., 2001, p. 88). In conflict situations with peers, the child may activate these cognitive schemas which give rise to behavior that makes the child a target for bullying (Batanova & Loukas, 2014). Thus, we hypothesize that for children who have relatively lower levels of inhibitory control, harsh parenting will predict being victimized (*H2*). We take an exploratory approach to investigate how children's inhibitory control problems may moderate the relationship between harsh parenting and showing perpetrator‐target behavior. As aforementioned, perpetrator‐targets share characteristics with both perpetrators as well as targets. A combination of the above explanations could illustrate how the effects of harsh parenting on the perpetrator‐target role can differ by inhibitory control problem levels. However, given the lack of theoretical foundations for how inhibitory control might moderate linkages between harsh parenting and perpetrator‐target behavior, no a priori hypotheses were formulated.

### 1.2 The importance of differentiating between parent and child sex

Most of the studies that explored the effects of harsh parenting on children's bullying involvement focused on the role of mothers (e.g., Barker et al., 2008) or “primary caregivers” (e.g., Fujikawa et al., 2018). Given the vast amount of literature on the importance of studying both paternal and maternal parenting effects on child outcomes (Volling et al., 2019), we explore the impact of mothers’ and fathers’ harsh parenting on bullying involvement. Studies find that same‐sex modeling (when gender normative) is more common than opposite‐sex modeling (Bandura, 1989). Despite changing gender roles in modern society (Guerrero & Schober, 2020), for boys it is traditionally more acceptable to model hostile behavior of their father, while for girls, aggressive behavior is less accepted (Perren & Alsaker, 2006). Consequently, we expect that same‐sex modeling will be more common, but solely for father‐son dyads.

### 1.3 The current study

We studied to what extent linkages between harsh parenting and bullying involvement differ by child inhibitory control problem levels. We hypothesized that harsh parenting would more likely lead to being the target of bullying when children have lower level problems with inhibitory control, and that harsh parenting would more likely lead to perpetrator behavior when children have higher levels of inhibitory control problems. For perpetrator‐targets, we took an exploratory approach. Furthermore, we explored the impact of parent and child sex on these linkages. We expected to find the strongest linkages between paternal harsh parenting and boys’ bullying involvement, as compared to maternal harsh parenting and girls’ bullying involvement.

## 2 METHODS

### 2.1 Procedure, design, and study population

This project is embedded in the Generation R Study, a multi‐informant population‐based prospective cohort in Rotterdam, the Netherlands (Kooijman et al., 2016). Recruited through midwives and obstetricians, pregnant women with an expected delivery date between April 2002 and January 2006, living in the study area were invited to participate. The Medical Ethical Committee of the Erasmus University Medical Center in Rotterdam approved the study, in accordance with the Declaration of Helsinki of the World Medical Association. Parents provided written informed consent for their own participation and on behalf of their child.

Within Generation R, there were 9749 live births registered, from which 7893 children participated in the study after birth. Teachers reported on bullying involvement at age six for 4282 children. From this sample, we had data on harsh parenting at age three for 2995 mothers and 2383 fathers, and data on inhibitory control at age four for 2994 children. Children with missing data on either maternal harsh parenting (*N* = 53), paternal harsh parenting (*N* = 505), or inhibitory control (*N* = 184) were excluded, leaving a complete‐case sample of 2131 families with information on all measures of harsh parenting, inhibitory control, and bullying involvement. We compared the complete‐case sample (*N* = 2131) with the sample that also allowed missings on harsh parenting and inhibitory control (*N* = 4282). Families in the latter sample had a lower household income and included younger and lower educated mothers and fathers than the complete‐case sample (all estimates *p* < .001). See Figure S1 in the Supporting Information Appendix for details on attrition.

### 2.2 Instruments

#### 2.2.1 Harsh parenting

Mothers and fathers self‐reported on six harsh parenting items from the Parent‐Child Conflict Tactics Scale (CTSPC; Straus et al., 1998) at child age three (in months, *M* (*SD*) = 36.44 (1.05), range 33.98–47.25). Ten items were selected from the original CTSPC scale (Jansen, Raat, et al., 2012), including items from the Nonviolent Discipline scale (four items), the Psychological Aggression scale (four items), Minor Assault scale (one item), and Severe Assault scale (one item). From the original Minor Physical Assault scale, three items were excluded from the Generation R study (e.g., “hit child on the bottom with something like a belt, stick or some other object”), as they are illegal practices in the Netherlands (Knox, 2010). Furthermore, an age‐inappropriate question (“said you would kick child out of the house”) from the Psychological Aggression scale was excluded (Straus et al., 1998). Cotter et al. (2018) found preliminary support for the CTSPC scale's reliability and convergent validity. Parents rated the prevalence of harsh parenting in the past two weeks on a 6‐point scale from “never” to “five times.” Due to the low frequency of responses in the “twice,” “three times,” “four times,” and “five times” categories, we combined these into “twice or more” for analyses (Jansen, Raat, et al., 2012). An exploratory factor analysis on the disciplining practices assessed in the Generation R cohort sample (Jansen, Raat, et al., 2012) showed that six out of the ten assessed items loaded onto the harsh discipline construct for both mothers and fathers. The present study included those six items: (1) I shook my child, (2) I shouted or screamed angrily at my child, (3) I called my child names, (4) I threatened to give a slap but I didn't do it, (5) I angrily pinched my child's arm, and (6) I called my child stupid or lazy or something like that. A continuous weighted scale was created to sum up the frequency of the six harsh parenting practices, ranging from 0 to 12, which allowed for two missing items per case. A higher score indicates more frequent and varied harsh parenting.

#### 2.2.2 Bullying involvement

Teachers reported on their student's bullying involvement at age six (in months, *M* (*SD*) = 77.38 (13.76), range 51.60–119.70) in elementary schools in Rotterdam during the past three months. The measure included four bullying and four victimization items (Perren & Alsaker, 2006). Out of the eight items, four referred to physical, verbal, relational, or material bullying, and four items analogously referred to victimization. Physical bullying was defined as hitting, kicking, pinching, or biting. Verbal bullying was defined as teasing, laughing at, or calling names. Relational bullying was defined as excluding other children, and material bullying was defined as hiding or breaking the belongings of another child. Teachers rated the items for each child on a four‐point scale indicating bullying involvement: “almost never,” “around one‐to‐three times per month,” “around one‐to‐two times per week,” or “more than twice per week.” In line with Perren and Alsaker's (2006) definition, we classified children as perpetrators when they bullied others at least “one‐to‐three times per month” on at least one bullying item and when they had not been victimized on any of the four items in the past three months. Children were classified as targets of bullying when they were victimized at least “one‐to‐three times per month” on at least one item and when they never bullied others on any item. Children were classified as perpetrator‐targets when they bullied others at least “one‐to‐three times per month” on at least one item and when they also were victimized at least “one‐to‐three times per month” on at least one item. Children were classified as uninvolved in bullying when they have never bullied others and were never victimized on any item (Jansen, Verlinden, et al., 2012). In the current sample, the items measuring bullying behavior and victimization had Cronbach's alphas of .73 and .64, respectively. The relatively low levels of internal consistency for both the bullying behavior and victimization items were to be expected as each item taps into different types of bullying behavior and victimization.

#### 2.2.3 Inhihbitory control

Inhibitory control was measured using the Behavior Rating Inventory of Executive Function in Preschool Children (BRIEF‐P, Isquith et al., 2004) when children were four years old (in months, *M* (*SD*) = 48.49 (0.96), range 47.05–60.77). The Inhibit scale of the BRIEF‐P taps into parental expectations of their child's ability to modulate and inhibit inappropriate responses, actions, and behavior (Isquith et al., 2004). Mothers rated 16 items using a 3‐point scale, ranging from “never or not at all” to “often or clearly.” We created a weighted, continuous scale ranging from 0 to 48, which allowed for two missing items per case. A higher score indicates increased problems in inhibitory control. The Inhibit scale in BRIEF‐P has been shown to have good internal consistency and convergent validity (Duku & Vaillancourt, 2014). In the current sample, the scale had good internal consistency with Cronbach's alpha being .88.

#### 2.2.4 Covariates

Based on previous studies showing associations with harsh parenting (Jansen, Raat, et al., 2012) and bullying involvement (Jansen, Verlinden, et al., 2012), we included child sex and age (obtained from birth records) as covariates. Information on highest attained maternal and paternal educational level, household income, and parents’ and their parents’ country of birth, which were used to define parents’ ethnicity, were all collected via prenatal questionnaires.

### 2.3 Analyses

All analyses were carried out using IBM SPSS Statistics version 25 (IBM Corp. Released, 2017). Using multiple imputations with fully conditional specification, we imputed missing data on mothers’ and fathers’ educational level and household income. Missing values were imputed based on the observed values of the independent variables, covariates measured prenatally, marital status measured prenatally, as well as the socioeconomic covariates measured at age three and six years. Marital status was added to the step of the multiple imputations to improve the imputed values for socioeconomic covariates. Based on the highest percentage of missing values (i.e., paternal educational level, 31% missingness), we generated 31 datasets (White et al., 2011). We report the pooled results for all regression analyses.

All main analyses were conducted on the complete‐case sample consisting of 2131 children, for whom we had complete data on bullying involvement, maternal and paternal harsh parenting, inhibitory control, and imputed data on covariates. We performed multinomial logistic regression analyses with a stepwise approach to investigate our research aims. We mean‐centered all continuous variables. By taking a stepwise approach, we initially modeled the relations of mothers’ and fathers’ harsh parenting with the odds of being a perpetrator, a target, and a perpetrator‐target, with the reference category being uninvolved in bullying. These associations were adjusted for covariates. Only covariates that meaningfully changed the effect estimates were included in the model. As parents’ ethnicity did not change the effect estimates, we removed these variables from our analyses. In the second model, we added child inhibitory control. In the third model, we added an interaction between mothers’ harsh parenting and child sex. In the fourth model, we tested the interaction between mothers’ harsh parenting and inhibitory control. In the fifth model, we included three separate two‐way interactions, and the three‐way interaction between mothers’ harsh parenting, child sex, and inhibitory control. In the sixth, seventh, and eighth models, we tested the same two‐way and three‐way interactions with fathers’ (instead of mothers’) harsh parenting as in the third, fourth, and fifth models respectively. To elaborate on significant interactions, we conducted additional multinomial logistic regression analyses where the moderator(s) were centered at ± 1 *SD* (Jaccard, 2001).

## 3 RESULTS

### 3.1 Population characteristics

Child (50.6% boys) and family characteristics are presented in Table 1. We classified 12.9% of the children as perpetrators, 4.2% as targets of bullying, 10.7% as perpetrator‐targets, and 72.2% as uninvolved. Fewer girls than boys were classified as perpetrators, targets, and perpetrator‐targets. Girls experienced less harsh parenting than did boys. Based on the highest 20% of the harsh parenting scale score (Jansen, Raat, et al., 2012), 27.4% of boys and 23.6% of girls experienced harsh parenting by one parent, and 13.1% of boys and 6.5% of girls experienced harsh parenting by both parents. Correlations between study variables can be found in the Supporting Information Appendix (Table 2). We investigated the associations between harsh parenting, inhibitory control, and the odds of children being involved in bullying as perpetrators, targets, and perpetrator‐targets, with the reference category being *uninvolved* in bullying. The results of the models we discuss below are adjusted for child and family covariates. In the Supporting Information Appendix (Tables 3–6) we report all models. There we also report sensitivity and nonresponse analyses.

**Table 1:** Descriptive statistics of sample characteristics (*N* = 2131)

		Girls	Boys	
**Child characteristics**		*N* (%)	*N* (%)	Total (%)
Bullying involvement	Uninvolved	813 (77.21)	725 (67.25)	1538 (72.17)
	Perpetrator	118 (11.21)	157 (14.56)	275 (12.90)
	Target of bullying	36 (3.42)	55 (5.10)	91 (4.27)
	Perpetrator‐Target	86 (8.17)	141 (13.08)	227 (10.65)
		*M* (*SD*)^a^	Min‐Max	
Inhibitory control problems	Girls	21.18 (4.36)	16‐46	
	Boys	22.88 (5.43)	16‐46	
**Family characteristics**		*M* (*SD*)^a^	Min–Max	*N* (%)
Maternal harsh parenting score	Girls	1.89 (1.63)	0–12	1053
	Boys	2.18 (1.91)	0–12	1078
Paternal harsh parenting score	Girls	1.58 (1.64)	0–12	1053
	Boys	2.06 (1.87)	0–12	1078
Parent age	Mothers	31.99 (4.26)	18.20–46.34	2131
	Fathers	34.17 (5.06)	19.37–57.23	1982
		**Mothers**	**Fathers**	
		*N* (%)	*N (%)*	
Ethnicity	Dutch	1553 (72.87)	1571 (73.72)	
	Other Western	190 (8.92)	134 (6.29)	
	Non‐Western	385 (18.07)	406 (19.05)	
	Missing	3 (0.14)	20 (0.94)	
Education level	Low^b^	55 (2.58)	55 (2.58)	
	Intermediate^c^	729 (34.21)	570 (26.75)	
	High^d^	1293 (60.67)	982 (46.08)	
	Missing	54 (2.53)	524 (24.58)	
		*N* (%)		
Household income	< €1200^e^	77 (3.61)		
	>€1200 and <€2000	251 (11.78)		
	>€2000^f^	1454 (68.23)		
	Missing	349 (16.37)		

^a^ Mean and standard deviations.

^b^ Low educational level refers to no or primary education.

^c^ Intermediate educational level refers to secondary or vocational education.

^d^ High educational level refers to Bachelor's degree, university‐level education.

^e^ Income below social security level.

^f^ Income higher than modal income.

### 3.2 The odds of being a perpetrator

Among direct associations, the odds of being a perpetrator were associated with fathers’ harsh parenting (odds ratio [OR] = 1.10, 95% confidence interval [CI] = 1.02–1.18) and inhibitory control (OR = 1.05, 95% CI = 1.02–1.07), but not with mothers’ harsh parenting. Inhibitory control did not moderate the associations between harsh parenting and being a perpetrator, and neither did child sex. No three‐way interactions between harsh parenting, inhibitory control, and child sex were found.

### 3.3 The odds of being a target of bullying

The odds of being a target of bullying were not directly associated with harsh parenting, nor with inhibitory control. While inhibitory control moderated the relations between mothers’ (but not fathers’) harsh parenting and the odds of being a target of bullying (mothers: OR = 0.97, 95% CI = 0.94–0.99; fathers: OR = 0.99, 95% CI = 0.97–1.02), child sex was not a significant moderator. The three‐way interaction between mothers’ (but not fathers’) harsh parenting, inhibitory control, and child sex was significantly associated with the odds of being a target of bullying (mothers: OR = 1.06, 95% CI = 1.01–1.12; fathers: OR = 1.04, 95% CI = 0.99–1.10). The graphical representation of how the association between mothers’ harsh parenting (per point increase) and the odds of being a target differs by the three inhibitory control groups per child sex is presented in Figure S2 in the Supporting Information Appendix. To investigate how the magnitude and direction of the moderation of mothers’ harsh parenting by inhibitory control varied by child sex, we probed the three‐way interaction using two approaches, namely by analyzing simple slopes and by using the Johnson‐Neyman technique (Bauer & Curran, 2005) (findings for the latter can be found in Supporting Information Appendix I). Simple slopes analyses revealed conditional associations between mothers’ harsh parenting and victimization (Table 6 in Supporting Information Appendix). For boys with *lower‐*level problems with inhibitory control (1 *SD* below the mean), mothers’ harsh parenting was related to an *increase* in the odds of being a target (OR = 1.25, 95% CI = 1.02–1.54). For boys with *higher‐*level problems with inhibitory control (1 *SD* above the mean), mothers’ harsh parenting was related to a *decrease* in the odds of being a target (OR = 0.70, 95% CI = 0.53–0.92). For girls, mothers’ harsh parenting was not related to being a target of bullying (OR_lower inhibitory control problems_ = 0.92, 95% CI = 0.65–1.29; OR_higher inhibitory control problems_ = 0.99, 95% CI = 0.78–1.24).

### 3.4 The odds of being a perpetrator‐target

The odds of being a perpetrator‐target were not directly associated with harsh parenting, but they were significantly associated with inhibitory control (OR = 1.07, 95% CI = 1.04–1.10). Inhibitory control did not moderate the relation between harsh parenting and the odds of being a perpetrator‐target. Child sex moderated the associations between mothers’ (but not fathers’) harsh parenting and the odds of being a perpetrator‐target (mothers: OR = 1.19, 95% CI = 1.03–1.39; fathers: OR = 0.95, 95% CI = 0.81–1.12). Moreover, the three‐way interaction between mothers’ (but not fathers’) harsh parenting, inhibitory control, and child sex was associated with the odds of being a perpetrator‐target (mothers: OR = 1.04, 95% CI = 1.01–1.07; fathers: OR = 1.00, 95% CI = 0.96–1.03). The graphical representation of the relation between mothers’ harsh parenting (per point increase) and the odds of being a perpetrator‐target by the three different inhibitory control problem groups per child sex can be found in the Supporting Information Appendix (Figure S3). To explore how the magnitude and direction of the moderation of the association between mothers’ harsh parenting and the odds of being a perpetrator‐target by inhibitory control varied by child sex, we analyzed simple slopes and regions of significance (findings for the latter can be found in the Supporting Information Appendix I). Simple slope analyses revealed that for girls with *higher* level (but not with *lower* level) inhibitory control problems, mothers’ harsh parenting was significantly associated with an *increased* odds of being a perpetrator‐target (OR_higher inhibitory control problems_ = 1.21, 95% CI = 1.05–1.40; OR_lower inhibitory control problems_ = 1.02, 95% CI = 0.83–1.25). For boys, mothers’ harsh parenting was not significantly associated with the odds of being a perpetrator‐target (OR_lower inhibitory control problems_ = 1.09, 95% CI = 0.93–1.28; OR_higher inhibitory control problems_ = 0.92, 95% CI = 0.82–1.03).

## 4 DISCUSSION

We investigated whether the relations between maternal and paternal harsh parenting and three bullying involvement roles (perpetrator, target, perpetrator‐target) differed by child inhibitory control and sex. Partially supporting our hypotheses, our results show that fathers' harsh parenting increased the odds of being a perpetrator unconditionally of inhibitory control, while the relations between mothers’ harsh parenting and the odds of being a target and a perpetrator‐target differed by child inhibitory control and sex.

### 4.1 Perpetrator behavior

Fathers’ harsh parenting increased the odds of being a perpetrator, but we did not find this relation for mothers. Our findings are consistent with studies that reported relations between negative and harsh parenting and increased child aggression and bullying (de Vries et al., 2018; Nocentini et al., 2019), as well as studies that found effects for fathers’ (but not mothers’) harsh parenting on aggression (Chang et al., 2003) and cyberbullying (Zurcher et al., 2018). The finding that inhibitory control problems increased the odds of being a perpetrator is consistent with previous reports on inhibitory control being related to externalizing problems (Riggs et al., 2004) and aggression (Raaijmakers et al., 2008). Contrary to our expectations, inhibitory control did not moderate the relationship between harsh parenting and the odds of being a perpetrator. Our results imply that situations in which fathers exhibit harsh parenting are likely to be key socialization moments, in which children learn to model physically or verbally inappropriate behavior (Bandura, 1989; Perry et al., 2001).

The fact that we only found direct and unconditional linkages with fathers’, and not mothers’, harsh parenting, could reflect on the measurement of harsh parenting in this study. Our harsh parenting measure taps into the frequency of this behavior, with mothers reporting slightly more harsh parenting than fathers. If the frequency of harsh parenting was all that mattered, we would most likely have seen (more) unconditional associations for mothers’ harsh parenting. Our results therefore suggest that, in addition to frequency, other aspects of harsh parenting, such as its perceived justness, might contribute. In the study by Alampay et al. (2017), taking into account frequency, fathers’, but not mothers’, perceived justness of punishment was positively related to child‐reported aggression. This finding is in line with our rationale that children learn from their parents that hostility is appropriate when this is justified as such (i.e., the other party deserves it). Children's perception of the justness of parental harshness may vary by the parent's sex, which might lead children to process harsh parenting differently. Alampay et al.'s (2017) findings also suggest that children may perceive fathers’ harsh parenting as more just and deserved than that of mothers. This might explain why we only found unconditional effects for fathers’ harsh parenting and only on the likelihood of being a perpetrator.

### 4.2 Victimization

Fathers’ harsh parenting was unrelated to the odds of being a target of bullying. The relation between mothers’ harsh parenting and the odds of being a target of bullying varied by inhibitory control, albeit for boys only. In line with our hypothesis, our results showed that for boys with *lower*‐level inhibitory control problems, mothers’ harsh parenting *increased* the odds of being a target of bullying. Unexpectedly, our results also showed that for boys with *higher‐*level inhibitory control problems, mothers’ harsh parenting *decreased* the odds of being a target of bullying. This suggests that higher‐level inhibitory control problems “buffer” against the negative influence of mothers’ harshness on victimization. Children who have higher levels of inhibitory control problems deal with parental harshness by an outward expression of their frustrations (e.g., Ziv et al., 2013), which may help taking up an assertive stance against peers. Assertive behavior might help boys defend themselves from aggressive peers, as impulsive and hostile behavior exhibited by boys is more accepted by peers than the same behavior shown by girls (Perren & Alsaker, 2006; Vaillancourt & Hymel, 2006).

Previous studies, albeit in different domains, have also found protective effects of higher inhibitory control problems levels/low levels of inhibitory control. Sette et al. (2018) studied relations between shyness and social and school adjustment and found that among children with lower inhibitory control levels, shyness was positively associated with regulated school behavior. The authors argued that higher inhibitory control may contribute to children's behavioral rigidity, making them be perceived as less well behaved, which in turn may be a risk for adjustment difficulties. Similarly, Brooker et al. (2016) found a positive relation between social anxiety and socially anxious behavior in children who had higher inhibitory control. These findings suggest that excessive behavioral inhibition can be detrimental in certain contexts (e.g., shyness, social anxiety), while the opposite pattern may be found when children have more inhibitory control problems. Our findings support both notions: (1) mothers’ harsh parenting increased victimization for boys with lower‐level inhibitory control problems, which might be due to excessive behavioral overcontrol and (2) mothers’ harsh parenting decreased victimization for boys with higher‐level inhibitory control problems. That said, we urge caution in the interpretation of these findings, as more studies are needed to replicate the results and investigate the mechanisms underlying the relations between harsh parenting and victimization.

### 4.3 Perpetrator‐target behavior

Although fathers’ harsh parenting was not related to the odds of being a perpetrator‐target, the relations between mothers’ harsh parenting and the odds of being a perpetrator‐target depended on children's inhibitory control and sex. We found variation in inhibitory control in the linkages between mothers’ harsh parenting and perpetrator‐target behavior for girls, but not for boys. Specifically, for girls with *higher* level inhibitory control problems, mothers’ harsh parenting was related to increased likelihood of being a perpetrator‐target. This finding suggests that girls who have higher level inhibitory control problems might deal with maternal harsh parenting by an outward expression of frustration, and internalize experiences from mothers’ harshness as cognitive schemas that might prompt them to “imitate” learnt hostile behavior. Impulsive and hostile behavior might be perceived by teachers and peers as less appropriate behavior for girls than boys (Isquith et al., 2004; Perry et al., 2001), and might therefore evoke counter‐responses by peers, which may maintain a perpetrator‐target status.

### 4.4 Strengths, limitations, and recommendations for future studies

The novelty of the present study lies in the core of our moderation models. We addressed a new perspective in the literature on bullying involvement by exploring whether the contribution of harsh parenting to different bullying involvement roles is conditioned by children's inhibitory control problems. Drawing on data derived from a population‐based multi‐informant study, we were able to eliminate single‐source bias. When interpreting the findings, however, one should keep in mind our study's shortcomings. First, theoretical work on the dual perpetrator‐target role suggests that the group of children who take up a perpetrator‐target role is heterogeneous (Sung et al., 2018). The operationalization we used in the current paper did not allow for this heterogeneity to come forward. Second, we reported low, but significant, odd ratios, which indicate weak associations between harsh parenting, inhibitory control, and bullying involvement. As our outcome is focused on child behavior at the school setting, while our predictors are more likely reflections on parent and child behavior at home, there might be individual differences in behavior due to the differing demands and environmental structures across these two settings (e.g., Arruda et al., 2020). Third, it is likely that parents underreported on harsh parenting. Thus, our findings may actually be underestimations of the associations between harsh parenting and bullying involvement. Fourth, our sample consisted of intact families with heterosexual parents only, which limits the generalizability of our findings to other family constellations. We recommend future studies to examine the relations between harsh parenting, inhibitory control, and bullying involvement in all types of contemporary families. Fifth, families are argued to be the main socializing agents in childhood (Maccoby, 1992). In this study, we focused on parents, as we did not have data on harsh parenting by other family members (e.g., grandparents). Hence, our findings may be less generalizable to families in which members other than the parents pose as children's main socialization role models. Sixth, although our study is longitudinal, we cannot infer causal relations. As data on inhibitory control were not available before the measure of harsh parenting, the possibility of reverse causality could not be excluded. Studies with repeated measures of harsh parenting and inhibitory control are needed to disentangle the temporality between these variables and obtain a better understanding of the interacting antecedents of targets’ and perpetrator‐targets’ behavior. Finally, characteristics other than inhibitory control could (also) moderate the relationships between harsh parenting and bullying involvement. A plausible moderator for future studies to explore is child temperament, as it taps into additional aspects of child reactivity and self‐regulation, and its measure includes, but is not limited to, inhibitory control (Putnam & Rothbart, 2006).

### 4.5 Implications for interventions

Our findings suggest that integrating parental components in anti‐bullying interventions are crucial for reducing bullying involvement. While not all anti‐bullying interventions include a parental component, information (e.g., booklets) for parents and parent‐teacher meetings are key components contributing to the reduction in bullying involvement (see meta‐analyses by Huang et al., 2019; Ttofi & Farrington, 2012). Embedding components on the effects of harsh parenting in interventions for parents‐to‐be or parents of toddlers might help reduce child maltreatment and milder forms of harshness. Providing mothers and fathers with such components might advance the program effects of VoorZorg (the Dutch Nurse‐Family Partnership program), an evidence‐based prevention program in the Netherlands that primarily targets child maltreatment in high‐risk families (Mejdoubi et al., 2015). Considering that the Nurse–Family Partnership aims to increase father involvement (Olds, 2008), making mothers and fathers aware of the detrimental impact of harsh parenting might contribute to the reduction of later social difficulties.

Universal anti‐bullying interventions are not effective for all children (Kaufman et al., 2018). Our findings reflect upon reciprocal processes between the child, family, and environment, and underscore taking a family system's perspective (Cox & Paley, 1997). Anti‐bullying interventions may therefore benefit from targeting the family as a system, within which individual subsystems (mother, father, child) and the dyadic interactions between the parents and the child are interlinked (Cox & Paley, 1997).

## 5 CONCLUSIONS

We explored the moderating role of children's inhibitory control problem levels in the relations between maternal and paternal harsh parenting and the likelihood of being a perpetrator, a target of bullying, and a perpetrator‐target, and to what extent these associations varied by child sex. Fathers’ harsh parenting was unconditionally associated with being a perpetrator, whereas the associations between mothers’ harsh parenting and the likelihood of being a target and a perpetrator‐target were conditioned upon child inhibitory control and sex. Our results highlight the importance of differentiating by parent and child sex, and by child inhibitory control to understand the intricate relationship between harsh parenting and bullying involvement. We encourage researchers to move beyond studying direct associations and to consider individual differences in how children process harsh parenting. Our findings suggest that even low frequency harsh parenting at preschool age contributes to bullying involvement later on. We recommend early prevention and intervention efforts to incorporate components into their programs on the harmful influence of parental harshness on children's social development.

## Supporting information

Supplementary information.

Supplementary information.

Supplementary information.

Supplementary information.

Supplementary information.

Supplementary information.

Supplementary information.

Supplementary information.

Supplementary information.

Supplementary information.

Supplementary information.

Supplementary information.

## Data Availability

Data can be obtained upon request. Requests should be directed toward the management team of the Generation R Study (secretariaat.genr@erasmusmc.nl), which has a protocol of approving data requests. Because of restrictions based on privacy regulations and informed consent of participants, data cannot be made freely available in a public repository.
