# Supplementary material for: The relation between harsh parenting and bullying involvement and the moderating role of child inhibitory control: A population‐based study
Source: Aggress Behav. 2021 Dec 16;48(2):141–51. doi: 10.1002/ab.22014 (PMC9299713; doi:10.1002/ab.22014)
Supplement: Supplementary file 1 — Supplementary information. [file AB-48-141-s008.docx]

**Supplementary Appendix I**

**Additional Analyses Conducted Using the PROCESS Macro in SPSS**

**Findings on Victimization**

We implemented the PROCESS macro in SPSS (Hayes, 2018) to use the Johnson-Neyman technique, which examines regions of significance for tests of interactions. In order be able to run the macro and to examine at which values of inhibitory control the association between harsh parenting and the odds of being a target of bullying was different between girls and boys, we made some concessions. First, as PROCESS is not compatible with a multiple imputed dataset, we ran the model of the complete case sample, in which we did not impute missings for our control variables. PROCESS uses listwise case deletion to deal with missing variables, therefore cases who had missings on the control variables were excluded from the model (excluded *N* = 479). Second, although PROCESS uses logistic regression to model a binary outcome variable, it uses ordinary least squared method for modeling count or ordinal outcome variables. As PROCESS is not able to run multinomial logistic regression analyses, we ran a binary logistic regression model, which included the outcome likelihood of being a target, with reference to being uninvolved. As such, our model for targets did not include children classified as perpetrators or perpetrator-targets (excluded *N* = 502). As a consequence of these two limitations, we modeled the likelihood of being a target (with reference to being uninvolved) in (1) a binary logistic regression analysis, as compared to multinomial regression, and in (2) a sample that was smaller (*N* = 1,150) than the sample that we used in the main analyses (*N* = 2,131). Therefore, we report the results binary logistic regression analyses on the likelihood of being a target as compared to being uninvolved. Our results reveal that the association between maternal harsh parenting and being a target was significantly different between boys and girls among those who had a score lower than 16.92 on inhibitory control problems. There were 21.6% of the children in this region of the distribution of inhibitory control. In addition, we found that the association between maternal harsh parenting and being a target was significantly different between boys and girls among those who had a score higher than 24.82 on inhibitory control problems. This region of the distribution of inhibitory control included 18.9% of the children.

**Findings on Perpetrator-Target Behavior**

For children classified as perpetrator-targets, we also run the PROCESS macro in SPSS to use Johnson-Neyman technique. We made similar concessions as reported above: We conducted a binary logistic regression model on the complete-case sample that excluded cases with a missing on control variables (excluded *N* = 515), and on the sample that did not include perpetrators and targets (excluded *N* = 366). We report the analyses on the likelihood of being a perpetrator-target (*N* = 1,250) as compared to being uninvolved. Our results revealed that the association between maternal harsh parenting and being a perpetrator-target was significantly different between boys and girls among those who had a score higher than 25.6 on inhibitory control problems. There were 20.1% of the children in this region of the distribution of inhibitory control.

**References**

Hayes, A. F. (2018). *Introduction to mediation, moderation, and conditional process analysis: A regression-based approach* (2^nd^ Ed.). The Guilford Press.
