# Supplementary material for: The relation between harsh parenting and bullying involvement and the moderating role of child inhibitory control: A population‐based study
Source: Aggress Behav. 2021 Dec 16;48(2):141–51. doi: 10.1002/ab.22014 (PMC9299713; doi:10.1002/ab.22014)
Supplement: Supplementary file 2 — Supplementary information. [file AB-48-141-s007.docx]

**Supplementary Appendix II**

**Non-Response Analyses**

We compared child characteristics across the complete-case sample (*N* = 2,131) and the sample that allowed missings on harsh parenting and inhibitory control (*N* = 4,282). The distribution of boys versus girls was comparable across the two samples (complete-case sample: 50.6% boys, 49.9% girls; sample with missings on harsh parenting and inhibitory control: 51.3% boys, 48.7% girls). The sample with missings contained a higher percentage of children involved in bullying than the complete case sample. The complete-case sample included 72.2% children uninvolved in bullying, 12.9% children classified as pure perpetrators, 4.3% children classified as pure targets, and 10.7% classified as perpetrator-targets. The sample with missings included 60.5% children uninvolved in bullying, 18.5% children classified as pure perpetrators, 4.8% children classified as pure targets, and 16.7% children classified as perpetrator-targets. Furthermore, children in the sample that allowed missings on harsh parenting and inhibitory control were significantly older (*M* = 78.48, *SD* = 14.00) than the children in the complete-case sample (*M* = 77.38, *SD* = 13.76; *p* = .010).

To examine non-response patterns with regards to the availability or absence of teacher reports on bullying involvement, we compared harsh parenting, child inhibitory control, parent’s age, education, and income in the complete-case sample (*N* = 2,131) with the sample that allowed children with missing data on bullying involvement (*N* = 3,441). We found no differences between the two samples in terms of harsh parenting, inhibitory control, parent age, or household income. Furthermore, we found that children with no data on bullying involvement had parents with slightly higher educational levels (*M_mother_* = 4.03, *M_father_* = 4.00, *p* < .001) than parents (*M_mother_* = 3.84, *M_father_* = 3.82), whose children had teacher reports on bullying involvement. Therefore, the results of this non-response analyses show that our sample consisted of relatively lower educated parents than the sample that did not have data on bullying involvement.
