# Supplementary material for: The relation between harsh parenting and bullying involvement and the moderating role of child inhibitory control: A population‐based study
Source: Aggress Behav. 2021 Dec 16;48(2):141–51. doi: 10.1002/ab.22014 (PMC9299713; doi:10.1002/ab.22014)
Supplement: Supplementary file 3 — Supplementary information. [file AB-48-141-s011.docx]

**Supplementary Appendix III**

**Sensitivity Analyses**

With three sensitivity analyses, we tested whether the associations between maternal and paternal harsh parenting and bullying involvement are robust with regards to the availability or absence of data on either maternal harsh parenting, paternal harsh parenting, or inhibitory control. First, we compared the associations with maternal harsh parenting (adjusted for covariates) in a complete-case sample (*N* = 2,131) versus a sample that includes cases with missing father report (*N* = 2,636). Second, we compared the associations of paternal harsh parenting (adjusted for covariates) with bullying involvement in a complete-case sample (*N* = 2,131) versus a sample that included cases with missing mother report (*N* = 2,184). Third, we compared the associations of maternal and paternal harsh parenting with bullying involvement in a complete-case sample (*N* = 2,131) versus a sample that included cases with missing inhibitory control (*N*= 2,315). The results in these three sensitivity analyses were like the original analysis, suggesting that the associations between harsh parenting and bullying involvement were robust with regards to the availability versus absence of data on maternal and paternal harsh parenting, and inhibitory control.
