# Supplementary material for: The relation between harsh parenting and bullying involvement and the moderating role of child inhibitory control: A population‐based study
Source: Aggress Behav. 2021 Dec 16;48(2):141–51. doi: 10.1002/ab.22014 (PMC9299713; doi:10.1002/ab.22014)
Supplement: Supplementary file 4 — Supplementary information. [file AB-48-141-s002.docx]

**Supplementary Figure Legends**

Figure 2. The association between maternal harsh parenting (per point increase) and the odds of being a target of bullying, presented for low (-1 *SD*), average, and higher (+1 *SD*) inhibitory control problem levels for boys and girls. Figure 3. The association between maternal harsh parenting (per point increase) and the odds of being a perpetrator-target, presented for low (-1 *SD*), average, and higher (+1 *SD*) inhibitory control problem levels for boys and girls.
