# Supplementary material for: The relation between harsh parenting and bullying involvement and the moderating role of child inhibitory control: A population‐based study
Source: Aggress Behav. 2021 Dec 16;48(2):141–51. doi: 10.1002/ab.22014 (PMC9299713; doi:10.1002/ab.22014)
Supplement: Supplementary file 5 — Supplementary information. [file AB-48-141-s004.docx]

| *Table 2. Correlations between study variables.* | | | | | | | | | | |
| --- | --- | --- | --- | --- | --- | --- | --- | --- | --- | --- |
|  | 1. | 2. | 3. | 4. | 5. | 6. | 7. | 8. | 9. | 10. |
| 1. Maternal HP | - | .38*** | .23*** | .06 | -.06 | -.08* | -.13*** | .07* | .01 | .12*** |
| 2. Paternal HP | .40*** | - | .18*** | .02 | .03 | .02 | -.07* | .05 | .02 | .04 |
| 3. Inhibitory control problems | .28*** | .22*** | - | .04 | -.06 | -.03 | -.08** | .12*** | .08* | .19*** |
| 4. Child age | .03 | .01 | .09** | - | -.07* | -.04 | -.05 | -.06 | .06 | .08* |
| 5. Maternal education | -.13*** | -.06 | -.20*** | -.06* | - | .50*** | .38*** | -.03 | -.03 | -.10** |
| 6. Paternal education | -.14*** | -.05 | -.20*** | -.06 | .59*** | - | .32*** | -.06 | -.10* | -.08 |
| 7. Household income | -.11*** | -.01 | -.13*** | -.01 | .44*** | .34*** | - | -.06 | -.09* | -13*** |
| 8. Perpetrator status | .00 | .09** | .08* | .06 | -.05 | -.05 | .01 | - | . | . |
| 9. Target status | -.01 | -.02 | .03 | .10** | -.06 | -.01 | -.05 | . | - | . |
| 10. Perpetrator-target status | .05 | .11** | .18*** | .05 | -.12** | -.13*** | -.03 | . | . | - |

*Note.* HP = harsh parenting. The upper half of the table represents girls, the lower half of the table represents boys. Variables 1 through 7 are continuous variables for which we report Pearson correlations. Variables 8 through 10 are dichotomous variables with reference category being uninvolved children. We report point-biserial correlations between the dichotomous and continuous variables. * *p* < .05 (two-tailed); ** *p* < .01 (two-tailed); *** *p* < .001 (two-tailed).
