# Supplementary material for: The relation between harsh parenting and bullying involvement and the moderating role of child inhibitory control: A population‐based study
Source: Aggress Behav. 2021 Dec 16;48(2):141–51. doi: 10.1002/ab.22014 (PMC9299713; doi:10.1002/ab.22014)
Supplement: Supplementary file 6 — Supplementary information. [file AB-48-141-s010.docx]

| *Table 3. The associations between harsh parenting. inhibitory control, child sex, and the odds of being a perpetrator (N = 2,131).* | | | | | |
| --- | --- | --- | --- | --- | --- |
|  | Odds ratio (95 % confidence interval) | | | | |
|  |  |  |  |  |  |
| Independent variables | **Model 0** | **Model 1** | **Model 2** | **Model 3** | **Model 4** |
| Intercept | 0.14*** | 0.26*** | 0.09*** | 0.28*** | 0.29*** |
| Maternal HP | 1.01 (0.94-1.09) | 1.00 (0.92-1.08) | 0.97 (0.90-1.06) | 0.92 (0.84-1.02) | 0.99 (0.91-1.07) |
| Paternal HP | 1.12 (1.04-1.21)*** | 1.11 (1.03-1.20)** | 1.10 (1.02-1.18)* | 1.10 (1.02-1.18)** | 1.09 (1.01-1.18)** |
| Household income |  | 1.02 (0.94-1.10) | 1.02 (0.94-1.11) | 1.02 (0.94-1.11) | 1.01 (0.93-1.10) |
| Maternal education |  | 0.94 (0.81-1.09) | 0.95 (0.82-1.11) | 0.95 (0.82-1.11) | 0.96 (0.82-1.11) |
| Paternal education |  | 0.91 (0.79-1.04) | 0.92 (0.80-1.05) | 0.92 (0.80-1.05) | 0.92 (0.80-1.06) |
| Child age |  | 1.00 (0.99-1.01) | 1.00 (0.99-1.01) | 1.00 (0.99-1.01) | 1.00 (0.99-1.01) |
| Child sex |  | 0.71 (0.55-0.92)* | 0.75 (0.58-0.98)* | 0.75 (0.58-0.98)* | 0.75 (0.58-0.97)* |
| Inhibition |  |  | 1.05 (1.02-1.07)*** | 1.05 (1.02-1.07)*** | 1.05 (1.02-1.08)*** |
| Maternal HP × Child sex |  |  |  | 1.14 (0.98-1.32) |  |
| Maternal HP × Inhibition |  |  |  |  | 0.99 (0.98-1.01) |
| McFadden’s pseudo R^2^ | 0.01 | 0.02 | 0.03 | 0.03 | 0.03 |
|  |  | | |  |  |
| Independent variables | **Model 5** | **Model 6** | **Model 7** | **Model 8** |  |
| Intercept | 0.30*** | 0.28*** | 0.28*** | 0.29*** |  |
| Maternal HP | 0.95 (0.85-1.06) | 0.97 (0.90-1.06) | 0.97 (0.90-1.06) | 0.98 (0.90-1.06) |  |
| Paternal HP | 1.09 (1.01-1.18)* | 1.11 (1.01-1.22)* | 1.10 (1.01-1.18)* | 1.12 (1.02-1.24)* |  |
| Household income | 1.01 (0.93-1.10) | 1.01 (0.93-1.10) | 1.02 (0.93-1.10) | 1.01 (0.93-1.10) |  |
| Maternal education | 0.95 (0.82-1.11) | 0.95 (0.82-1.11) | 0.95 (0.82-1.11) | 0.95 (0.82-1.11) |  |
| Paternal education | 0.91 (0.79-1.05) | 0.92 (0.80-1.06) | 0.92 (0.80-1.06) | 0.91 (0.80-1.05) |  |
| Child age | 1.00 (0.99-1.01) | 1.00 (0.99-1.01) | 1.00 (0.99-1.01) | 1.00 (0.99-1.01) |  |
| Child sex | 0.72 (0.55-0.94)* | 0.76 (0.58-0.98)* | 0.75 (0.58-0.98)* | 0.74 (0.57-0.96)* |  |
| Inhibition | 1.04 (1.00-1.07)* | 1.05 (1.02-1.07)*** | 1.05 (1.02-1.07)*** | 1.03 (0.99-1.07) |  |
| Maternal HP × Child sex | 1.10 (0.94-1.29) |  |  |  |  |
| Maternal HP × Inhibition | 0.99 (0.98-1.01) |  |  |  |  |
| Inhibition × Child sex | 1.03 (0.98-1.09) |  |  |  |  |
| Maternal HP × Child sex × Inhibition | 1.02 (0.99-1.05) |  |  |  |  |
| Paternal HP × Child sex |  | 0.97 (0.84-1.12) |  | 0.94 (0.81-1.09) |  |
| Paternal HP × Inhibition |  |  | 1.00 (0.99-1.01) | 1.00 (0.98-1.02) |  |
| Inhibition × Child sex |  |  |  | 1.05 (0.99-1.11) |  |
| Paternal HP × Child sex × Inhibition |  |  |  | 1.01 (0.98-1.04) |  |
| McFadden’s pseudo R^2^ | 0.04 | 0.03 | 0.03 | 0.03 |  |

*Note.* HP = harsh parenting. * *p* < .05; ** *p* < .01; *** *p* < .001.
