# Supplementary material for: The relation between harsh parenting and bullying involvement and the moderating role of child inhibitory control: A population‐based study
Source: Aggress Behav. 2021 Dec 16;48(2):141–51. doi: 10.1002/ab.22014 (PMC9299713; doi:10.1002/ab.22014)
Supplement: Supplementary file 7 — Supplementary information. [file AB-48-141-s005.docx]

| *Table 4. The associations between harsh parenting, inhibitory control, child sex, and the odds of being a target of bullying (N = 2,131).* | | | | | |
| --- | --- | --- | --- | --- | --- |
|  | Odds ratio (95 % confidence interval) | | | | |
|  |  |  |  |  |  |
| Independent variables | **Model 0** | **Model 1** | **Model 2** | **Model 3** | **Model 4** |
| Intercept | 0.06*** | 0.07** | 0.03*** | 0.28* | 0.30* |
| Maternal HP | 1.00 (0.88-1.14) | 0.97 (0.85-1.10) | 0.95 (0.83-1.09) | 0.93 (0.79-1.09) | 0.99 (0.86-1.13) |
| Paternal HP | 1.01 (0.89-1.16) | 1.00 (0.87-1.14) | 0.99 (0.86-1.13) | 0.99 (0.86-1.13) | 0.97 (0.85-1.11) |
| Household income |  | 0.90 (0.81-1.01) | 0.91 (0.81-1.01) | 0.90 (0.80-1.01) | 0.89 (0.79-1.00)* |
| Maternal education |  | 0.95 (0.74-1.22) | 0.96 (0.75-1.23) | 0.96 (0.75-1.23) | 0.98 (0.76-1.26) |
| Paternal education |  | 0.95 (0.76-1.19) | 0.96 (0.77-1.20) | 0.96 (0.77-1.20) | 0.97 (0.77-1.21) |
| Child age |  | 1.02 (1.01-1.04)** | 1.02 (1.01-1.04)** | 1.02 (1.01-1.03)** | 1.02 (1.01-1.03)** |
| Child sex |  | 0.59 (0.38-0.90)* | 0.61 (0.39-0.95)* | 0.61 (0.40-0.95)* | 0.60 (0.39-0.94)* |
| Inhibition |  |  | 1.04 (0.99-1.08) | 1.04 (0.99-1.08) | 1.04 (0.99-1.08) |
| Maternal HP × Child sex |  |  |  | 1.06 (0.82-1.37) |  |
| Maternal HP × Inhibition |  |  |  |  | 0.97 (0.94-0.99)** |
| McFadden’s pseudo R^2^ | 0.01 | 0.02 | 0.03 | 0.03 | 0.03 |
|  |  | | |  |  |
| Independent variables | **Model 5** | **Model 6** | **Model 7** | **Model 8** |  |
| Intercept | 0.32* | 0.27* | 0.28* | 0.29* |  |
| Maternal HP | 0.98 (0.83-1.15) | 0.95 (0.83-1.09) | 0.95 (0.83-1.08) | 0.95 (0.83-1.08) |  |
| Paternal HP | 0.97 (0.85-1.12) | 0.95 (0.80-1.13) | 1.00 (0.87-1.14) | 0.99 (0.83-1.17) |  |
| Household income | 0.89 (0.79-1.00)* | 0.90 (0.81-1.01) | 0.90 (0.80-1.01) | 0.90 (0.81-1.01) |  |
| Maternal education | 0.99 (0.77-1.28) | 0.96 (0.75-1.23) | 0.96 (0.75-1.24) | 0.97 (0.76-1.24) |  |
| Paternal education | 0.95 (0.76-1.19) | 0.96 (0.77-1.20) | 0.96 (0.77-1.20) | 0.95 (0.76-1.19) |  |
| Child age | 1.02 (1.00-1.03)** | 1.02 (1.01-1.03)** | 1.02 (1.01-1.03)** | 1.02 (1.01-1.03)** |  |
| Child sex | 0.57 (0.36-0.89)* | 0.62 (0.40-0.96)* | 0.61 (0.39-0.95)* | 0.57 (0.36-0.89)* |  |
| Inhibition | 1.02 (0.96-1.07) | 1.04 (0.99-1.08) | 1.04 (0.99-1.08) | 1.02 (0.96-1.07) |  |
| Maternal HP × Child sex | 0.98 (0.75-1.28) |  |  |  |  |
| Maternal HP × Inhibition | 0.95 (0.92-0.98)* |  |  |  |  |
| Inhibition × Child sex | 1.07 (0.97-1.17) |  |  |  |  |
| Maternal HP × Child sex × Inhibition | 1.06 (1.01-1.12)* |  |  |  |  |
| Paternal HP × Child sex |  | 1.11 (0.86-1.43) |  | 1.01 (0.77-1.33) |  |
| Paternal HP × Inhibition |  |  | 0.99 (0.97-1.02) | 0.98 (0.95-1.01) |  |
| Inhibition × Child sex |  |  |  | 1.06 (0.97-1.16) |  |
| Paternal HP × Child sex × Inhibition |  |  |  | 1.04 (0.99-1.10) |  |
| McFadden’s pseudo R^2^ | 0.04 | 0.03 | 0.03 | 0.03 |  |

*Note.* HP = harsh parenting. * *p* < .05; ** *p* < .01; *** *p* < .001.
