# Supplementary material for: The relation between harsh parenting and bullying involvement and the moderating role of child inhibitory control: A population‐based study
Source: Aggress Behav. 2021 Dec 16;48(2):141–51. doi: 10.1002/ab.22014 (PMC9299713; doi:10.1002/ab.22014)
Supplement: Supplementary file 8 — Supplementary information. [file AB-48-141-s003.docx]

| *Table 5. The associations between harsh parenting, inhibitory control, child sex, and the odds of being a perpetrator-target (N = 2,131).* | | | | | |
| --- | --- | --- | --- | --- | --- |
|  | Odds ratio (95 % confidence interval) | | | | |
|  |  |  |  |  |  |
| Independent variables | **Model 0** | **Model 1** | **Model 2** | **Model 3** | **Model 4** |
| Intercept | 0.10*** | 0.22** | 0.05*** | 0.51 | 0.52 |
| Maternal HP | 1.10 (1.02-1.19)* | 1.06 (0.98-1.15) | 1.03 (0.95-1.12) | 0.96 (0.87-1.06) | 1.05 (0.96-1.15) |
| Paternal HP | 1.11 (1.02-1.20)* | 1.10 (1.01-1.19)* | 1.07 (0.99-1.16) | 1.07 (0.98-1.16) | 1.06 (0.98-1.16) |
| Household income |  | 0.99 (0.91-1.07) | 1.00 (0.92-1.08) | 0.99 (0.91-1.08) | 0.98 (0.91-1.07) |
| Maternal education |  | 0.84 (0.72-0.99)* | 0.86 (0.74-1.02) | 0.87 (0.74-1.02) | 0.87 (0.74-1.03) |
| Paternal education |  | 0.87 (0.76-1.01) | 0.88 (0.76-1.02) | 0.88 (0.76-1.02) | 0.89 (0.77-1.03) |
| Child age |  | 1.01 (1.00-1.02)* | 1.01 (1.00-1.02) | 1.01 (1.00-1.02) | 1.01 (1.00-1.02) |
| Child sex |  | 0.59 (0.44-0.79)*** | 0.65 (0.49-0.88)** | 0.63 (0.47-0.85)** | 0.65 (0.48-0.87)** |
| Inhibition |  |  | 1.07 (1.04-1.10)*** | 1.07 (1.04-1.10)*** | 1.07 (1.04-1.10)*** |
| Maternal HP × Child sex |  |  |  | 1.19 (1.03-1.39)* |  |
| Maternal HP × Inhibition |  |  |  |  | 0.99 (0.98-1.00) |
| McFadden’s pseudo R^2^ | 0.01 | 0.02 | 0.03 | 0.03 | 0.03 |
|  |  | | |  |  |
| Independent variables | **Model 5** | **Model 6** | **Model 7** | **Model 8** |  |
| Intercept | 0.52 | 0.49 | 0.50 | 0.50 |  |
| Maternal HP | 1.02 (0.91-1.14) | 1.03 (0.95-1.11) | 1.03 (0.95-1.11) | 1.03 (0.95-1.11) |  |
| Paternal HP | 1.06 (0.98-1.16) | 1.09 (0.99-1.20) | 1.07 (0.98-1.17) | 1.09 (0.98-1.22) |  |
| Household income | 0.99 (0.91-1.07) | 0.99 (0.91-1.07) | 0.99 (0.91-1.07) | 0.99 (0.91-1.07) |  |
| Maternal education | 0.88 (0.75-1.03) | 0.87 (0.74-1.02) | 0.87 (0.74-1.02) | 0.87 (0.74-1.02) |  |
| Paternal education | 0.87 (0.75-1.01) | 0.89 (0.77-1.03) | 0.89 (0.77-1.03) | 0.89 (0.77-1.03) |  |
| Child age | 1.01 (1.00-1.02) | 1.01 (1.00-1.02) | 1.01 (1.00-1.02) | 1.01 (1.00-1.02) |  |
| Child sex | 0.60 (0.44-0.82)** | 0.66 (0.49-0.89)** | 0.65 (0.49-0.88)** | 0.66 (0.49-0.90)** |  |
| Inhibition | 1.08 (1.04-1.12)*** | 1.07 (1.04-1.10)*** | 1.07 (1.04-1.10)*** | 1.06 (1.03-1.10)*** |  |
| Maternal HP × Child sex | 1.11 (0.94-1.32) |  |  |  |  |
| Maternal HP × Inhibition | 0.98 (0.97-1.00) |  |  |  |  |
| Inhibition × Child sex | 0.98 (0.92-1.04) |  |  |  |  |
| Maternal HP × Child sex × Inhibition | 1.04 (1.01-1.07)* |  |  |  |  |
| Paternal HP × Child sex |  | 0.95 (0.81-1.12) |  | 0.95 (0.80-1.13) |  |
| Paternal HP × Inhibition |  |  | 1.00 (0.99-1.01) | 1.00 (0.98-1.02) |  |
| Inhibition × Child sex |  |  |  | 1.02 (0.96-1.08) |  |
| Paternal HP × Child sex × Inhibition |  |  |  | 1.00 (0.96-1.03) |  |
| McFadden’s pseudo R^2^ | 0.04 | 0.03 | 0.03 | 0.03 |  |

*Note.* HP = harsh parenting. * *p* < .05; ** *p* < .01; *** *p* < .001.
