# Supplementary material for: The relation between harsh parenting and bullying involvement and the moderating role of child inhibitory control: A population‐based study
Source: Aggress Behav. 2021 Dec 16;48(2):141–51. doi: 10.1002/ab.22014 (PMC9299713; doi:10.1002/ab.22014)
Supplement: Supplementary file 9 — Supplementary information. [file AB-48-141-s009.docx]

| *Table 6. Conditional associations between maternal harsh parenting and the odds of being a target of bullying and a perpetrator-target at different levels of the two moderators (N = 2,131).* | | | |
| --- | --- | --- | --- |
|  |  | **Target of bullying** | **Perpetrator-target** |
| Child sex | Inhibitory control problem level | OR (95 % CI) | OR (95 % CI) |
| Boys | Low | 1.25 (1.02-1.54)* | 1.09 (0.93-1.28) |
| Boys | Average | 0.98 (0.83-1.15) | 1.02 (0.91-1.14) |
| Boys | High | 0.70 (0.53-0.92)* | 0.92 (0.82-1.03) |
| Girls | Low | 0.92 (0.65-1.29) | 1.02 (0.83-1.25) |
| Girls | Average | 0.96 (0.76-1.20) | 1.13 (0.99-1.29) |
| Girls | High | 0.99 (0.78-1.24) | 1.21 (1.05-1.40)** |
| *Note.* * *p* < .05; ** *p* < .01; *** *p* < .001. | | | |
