# Supplementary figures and images for: The relation between harsh parenting and bullying involvement and the moderating role of child inhibitory control: A population‐based study
Source: Aggress Behav. 2021 Dec 16;48(2):141–51. doi: 10.1002/ab.22014 (PMC9299713; doi:10.1002/ab.22014)

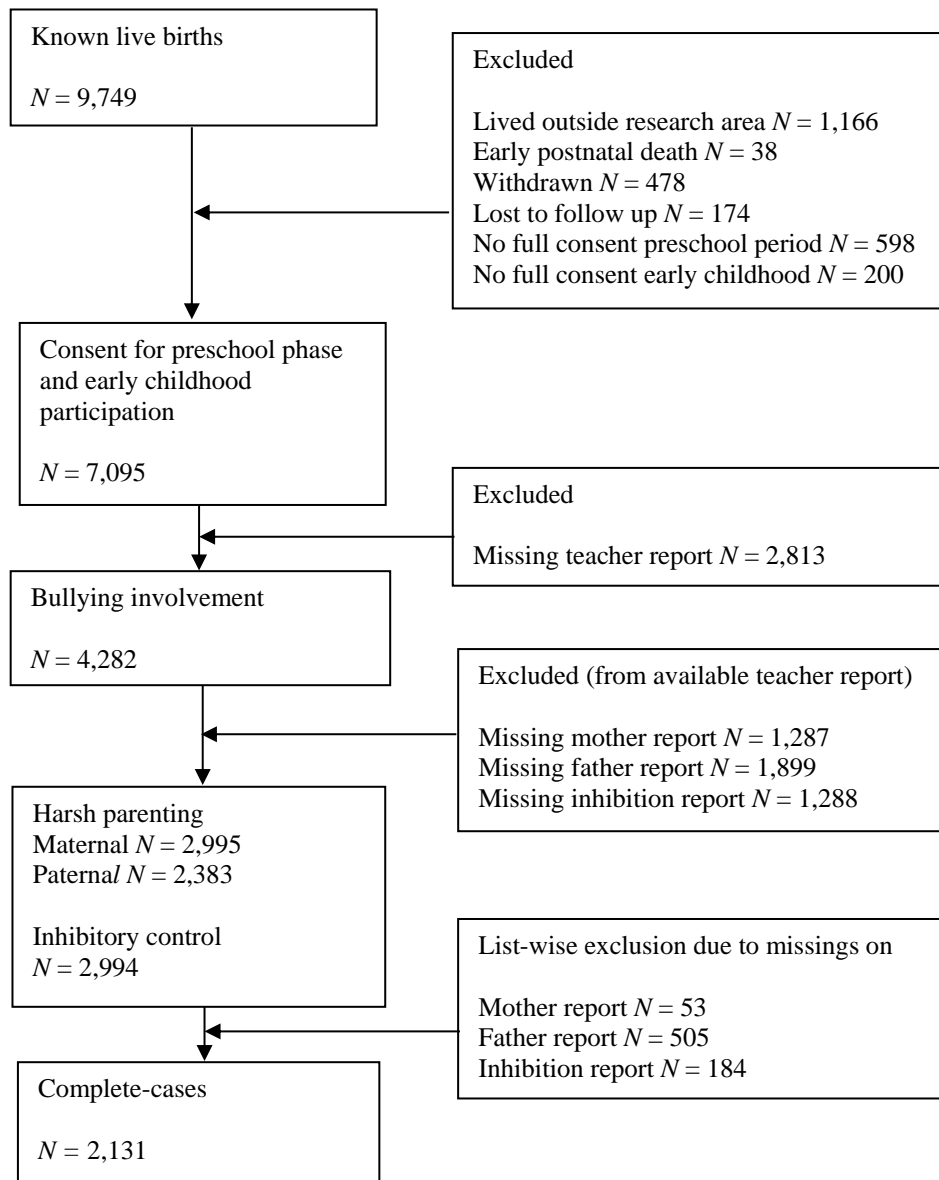

Figure 1. Flowchart of population attrition.

Supplement: Supplementary file 10 — Supplementary information. [file AB-48-141-s012.pdf]

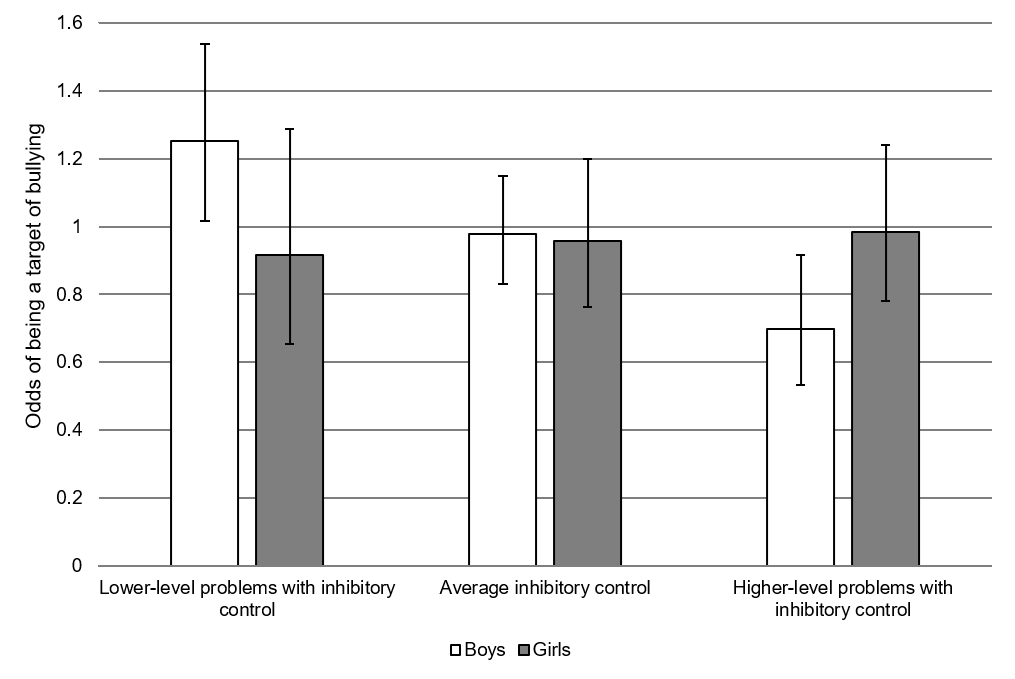

Supplement: Supplementary file 11 — Supplementary information. [file AB-48-141-s006.tif]

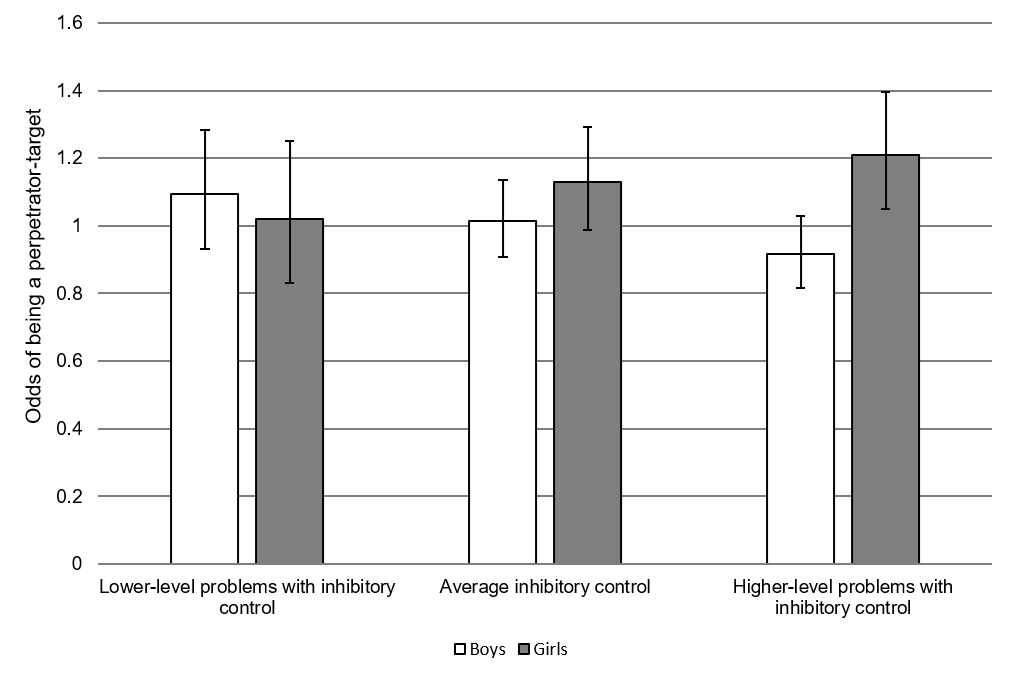

Supplement: Supplementary file 12 — Supplementary information. [file AB-48-141-s001.tif]
